# Inference of Microbial Recombination Rates from Metagenomic Data

**DOI:** 10.1371/journal.pgen.1000674

**Published:** 2009-10-02

**Authors:** Philip L. F. Johnson, Montgomery Slatkin

**Affiliations:** 1 Biophysics Graduate Group, University of California Berkeley, Berkeley, California, United States of America; 2 Integrative Biology, University of California Berkeley, Berkeley, California, United States of America; University of Arizona, United States of America

## Abstract

Metagenomic sequencing projects from environments dominated by a small number of species produce genome-wide population samples. We present a two-site composite likelihood estimator of the scaled recombination rate, *ρ = 2N_e_c*, that operates on metagenomic assemblies in which each sequenced fragment derives from a different individual. This new estimator properly accounts for sequencing error, as quantified by per-base quality scores, and missing data, as inferred from the placement of reads in a metagenomic assembly. We apply our estimator to data from a sludge metagenome project to demonstrate how this method will elucidate the rates of exchange of genetic material in natural microbial populations. Surprisingly, for a fixed amount of sequencing, this estimator has lower variance than similar methods that operate on more traditional population genetic samples of comparable size. In addition, we can infer variation in recombination rate across the genome because metagenomic projects sample genetic diversity genome-wide, not just at particular loci. The method itself makes no assumption specific to microbial populations, opening the door for application to any mixed population sample where the number of individuals sampled is much greater than the number of fragments sequenced.

## Author Summary

At a broad scale, the exchange of genetic material through homologous recombination (i.e. what happens in animals during sex) increases the potential rate of adaptation. Bacteria often reproduce clonally, without recombination, by making exact copies of their genomes, but they also have mechanisms analogous to sex that allow them to recombine sporadically. Despite microbes' critical role at the base of our world's ecosystem, microbiologists know surprisingly little about how microbes grow and evolve outside the laboratory. Metagenomic sequencing projects provide a means to sample the genetic diversity of natural microbial populations and have the potential to reveal much about the ecology and evolution of these populations. Here we present a novel method to estimate the recombination rate from metagenomic data, while explicitly allowing for imperfections such as sequencing error and missing data.

## Introduction

Microbial populations exchange homologous genetic material at different rates, dramatically affecting the evolutionary potential of the population. While basal mutation rates can be estimated via long-term within-laboratory evolution experiments [1], recombination rates are more difficult to infer because they require identification of multiple alleles at multiple loci in multiple individuals. Further, biogeographic barriers and interspecies interactions may lead to qualitatively different effects than growth in axenic laboratory culture, making determination of recombination rates in an organism's natural environment critical to accurate interpretation [2]. For the purpose of this study, we ignore the mechanism behind homologous recombination (i.e. transformation, transduction, or conjugation) and focus on its effect on genetic diversity.

Much research has investigated human recombination hotspots [3], yet almost nothing is known about variation in microbial recombination rates within a genome. In specific instances, however, studies have experimentally identified sequence motifs associated with recombination hotspots in some species of bacteria and yeast [4]. Mounting evidence suggests that regions known as CRISPR (Clusters of Regularly Interspaced Short Palindromic Repeats) form the basis of a bacterial immune system against phage in which chunks of the phage genome are inserted into the CRISPR region [5]. Thus a reasonable hypothesis would be that these regions or other regions with similar effect might recombine with greater frequency than the rest of the genome.

Inference of a genome-wide, fine-scale recombination map requires both extensive genome-wide sampling of the genetic diversity within the population of interest as well as an appropriate population genetic model, neither of which has been previously available for microbial populations. Microbial population surveys have primarily sequenced a small number of loci (“multi-locus sequence typing”) [6], which yield no information about variation in local recombination rate. Current methods tailored to microbial populations rely on low-power summary statistics [7],[8], heuristics instead of explicitly modeling the source of the recombining fragments [9], or parsimony based on manual inspection [10]. A few studies (e.g. [2],[11]) applied a more rigorous likelihood-based approach using a population genetic model ([12]; discussed more below), but these were still able to estimate only a genome-wide average rate of recombination.

Recently, large-scale metagenomic sequencing projects have begun to generate genome-wide population samples by sequencing random reads from a pool of DNA extracted from all microorganisms in a given environment. Projects that sample environments dominated by only a few microbial “species” are able to assemble near-complete genomes [13],[14], in which the constituent reads contain information about the genetic diversity in the population. Considering the large number of individuals in the sampled community relative to the number of reads sequenced, each read derives almost certainly from a different individual microorganism. With average read depths as high as ten [14], the resulting data hold rich potential for population genetic analysis [15],[16].

Given these data, we can make inferences about parameters such as mutation rate and recombination rate. In population genetic theory, the per-generation mutation rate, 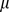, and per-generation recombination rate, 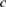, almost always appear in conjunction with the effective population size, 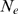, as the parameters 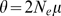 and 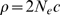. In our microbial context, we assume a single recombination event leads to the replacement of a short tract of sequence, creating two recombination breakpoints. A full likelihood method would yield maximal power by calculating the probability of observing the entire pattern of polymorphism across all samples, given the parameters 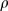 and 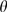. In practice, however, this approach is extremely computationally intensive [17], and even a recent breakthrough using a Markov chain Monte Carlo technique only extends full-likelihood to input data containing fewer than 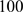 SNPs [18]. Instead, we follow the lead of previous researchers who sacrificed power for greater practicality by using a composite likelihood method [12],[19],[20] that approximates the true likelihood, as detailed in the Methods section.

However, metagenomic population samples differ from traditional population samples and, as a result, provide new challenges to estimating recombination. First, the sample size varies according to the read depth at a given location instead of being fixed across all loci. Second, the quality of each base call varies along each read, and the random nature of the metagenomic method prevents independent replication of the sampling and sequencing steps to confirm observed polymorphisms. Finally, linkage information is greatly reduced in that instead of the traditional approach of sampling the same individual at all loci, each fragment of DNA derives from a different individual. Depending on the sequencing technology and whether reads were sequenced in pairs, these data will reveal, at most, linkage within two reads of 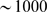 nucleotides that are separated by a distance generally less than 40 kilobases.

As high-throughput sequencing becomes ever cheaper, the number of projects producing this sort of data will only increase. The Human Microbiome Project (http://www.hmpdacc.org/) plans to perform metagenomic sequencing of microbes found at five sites around the body. A particularly intriguing future application will be to sequence mixtures of pathogens sampled from within a single infected human. These data, combined with the methods presented here, will allow inferences about the interplay between the immune response and recombination within pathogens.

## Methods

We start by deriving our two-locus composite likelihood estimator based on the idea of Hudson [19] and the estimator of McVean et al. [12] but now allowing for realistic amounts of missing data and sequencing error. Sequencing error probabilities are taken as given in the form of per-base quality scores. The resulting likelihood calculation becomes computationally infeasible on metagenomic-scale data, so we further describe several numerical approximations that make our implementation tractable. Finally we define a statistic to quantify the amount of missing data. This statistic will aid analysis and discussion of our estimator of 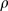.

### Composite-likelihood estimator

Our input data consist of a metagenomic assembly (i.e. alignment of reads to a scaffold), untrimmed FASTA sequences for the reads, quality scores for each base in each read and, if applicable, information about read pairs. We explicitly do not consider any uncertainty in either the assembly or in the quality scores for the practical reason that current assembly algorithms and base callers do not generate this information; however, in principle, our method could be extended to incorporate these sources of uncertainty. Given these data, we wish to estimate two population genetic parameters: 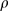 and 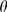.

**Figure 1:**
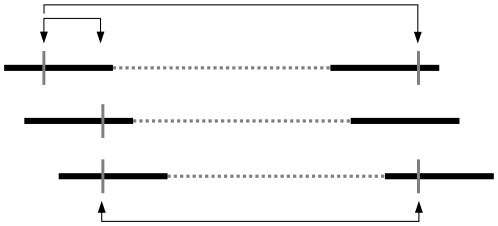
Cartoon metagenomic assembly. Three chromosomes, each with paired-end reads (bold horizontal lines) separated by a gap (dashed line). Assembly contains three polymorphic sites (vertical bars), which create three pairs of polymorphic sites (arrows). Note that our method actually uses all pairs of sites, not just polymorphic ones.

Following [12], we assume that each site in the assembly has at most two different nucleotides and arbitrary label these as zero and one. In the rare event that more than two distinct nucleotides are observed, then we again arbitrarily label them zero and one after first grouping the nucleotides into two categories: the most common nucleotide and everything else. In the case of a tie for the most common nucleotide, we pick one at random. Given this labeling, we can represent the state of a read at a given position by 0, 1 or ?, where the question mark represents missing data. Analogously, we represent the state of a single chromosome at two positions simultaneously: 00, 01, 10, 11, 0?, 1?, ?0, ?1 (ignoring ??, since this conveys no information). An example is given in Figure 1 and described below. Note that, in a metagenomic context, “a single chromosome” means that both nucleotides are either on the same read or on two paired reads. We assume that the total number of sequenced reads is much less than the total number of cells in the sampled environment such that the probability of two independent (unpaired) reads deriving from the same original cell/chromosome is essentially zero.

First we outline our notation more formally. The assembly, 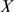, extends from position 1 to position 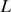 and contains information about both the content of the reads and their position. The set of quality scores, 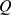, contains one quality score for each base in each read in the assembly. We assume Phred-calibrated quality scores [21], so any particular quality score, 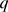, can be converted into an error probability, 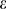, by means of the formula 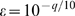. The configuration for a pair of sites, 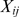 (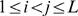), is a vector of eight numbers corresponding to the number of chromosomes observed in each of the eight states (00, 01, etc.). For example, in Figure 1 the configuration of the leftmost pair of polymorphic sites is {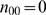, 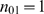, 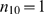, 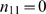, 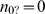, 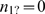, 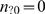, 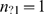}. In addition to the configuration at pair 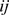, we also have the set of quality scores, 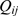 (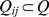).

We wish to calculate the likelihood of the observed data, 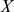, given the quality scores, 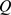, and the population genetic parameters of interest, 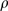 and 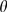. We approximate the true likelihood with the composite likelihood:
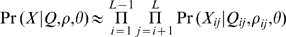
(1)in which the two-locus configurations are treated as though they were independent among pairs of sites. We take the mutation rate (and thereby 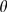) to be constant and independent across all sites in the assembly, conditional on the genealogy. However, the recombination rate between two sites 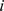 and 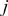 depends on their distance apart, 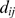, as measured by the number of nucleotides separating them. We model recombination in microbial populations as occurring via gene conversion with recombination tract lengths drawn from an exponential distribution [12],[22],[23]:
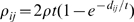
(2)where 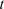 is the average length of the recombination tract. Theoretically 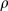 and 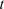 might be identifiable, but in practice our data are insufficient to separate them. Instead we fix 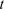 and estimate 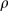, similar to the approach taken by McVean et al. [12]. Minor misspecification of 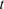 will simply rescale 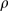, although major misspecification of 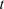 will also change the right-hand side of (2).

Now we turn to the likelihood of a single two-locus configuration. We first account for sequencing error by summing over all possibilities for the truth, 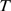:
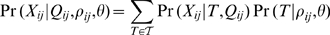
(3)where the sum iterates over all 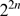 elements of the set of possible two-locus configurations, 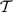, and 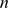 is the average number of reads at each site. The first term inside the sum is the error probability, while the second term is the two-locus likelihood without any error. We assume that sequencing errors cause a switch from 0 to 1 and vice versa:
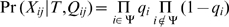
(4)where 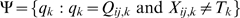 and the subscript 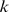 indexes the same position in the same read in the quality scores, the observation, and the truth. In other words, all mismatches between the truth and observed must be the result of an error, while all matches between the truth and the observed cannot have been caused by an error.

Next we account for missing data by summing over all possibilities for the unknown nucleotides in the complete configuration, 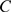:
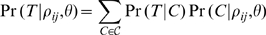
(5)where the sum iterates over all elements of the set of configurations compatible with the observed data, 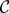 (i.e. those that satisfy the constraints 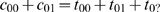, etc.). The first term inside the sum accounts for missing data, while the second term is the pure two-locus likelihood. If we treat the configurations 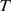 and 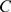 as a specific ordering of chromosomes, then this first term has a binary value of 1 for all configurations 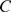 that match 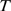 at non-missing positions and 0 otherwise. As a result of our definition for the set 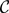, all configurations 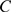 will match 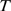 at non-missing positions, so the first term is always 1. We describe calculation of the second term in the next section below.

We arrive at the final composite likelihood equation by taking (1) and substituting in (3), (4) and (5), which leaves us with four nested products and sums of significant size as discussed below.

Now we wish to find maximum likelihood estimates to our parameters. Joint maximization of 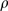 and 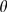 is computationally impractical. Instead, we perform a two-step estimation procedure in which we first estimate 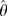 from single sites using a previously-developed method that correctly handles sequencing error [15] and then estimate 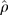 from pairs of sites by numerically maximizing (1) while holding 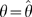.

### Two-locus complete likelihoods without error

We pre-calculate and store the two-locus likelihoods for all possible complete two-locus configurations without error (i.e. the second term in (5)) for a single sample size, 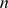, across a range of 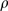 values and a single fixed 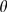 value. We generate this table of likelihoods by running a slightly modified version of the **complete** program from the LDhat package [12], which assumes a finite sites Jukes-Cantor style biallelic mutation model and uses the neutral coalescent-with-recombination importance sampling method of Fearnhead and Donnelly [24]. The original **complete** program computed likelihoods only for configurations in which both sites were observed to be polymorphic; our modification enables the calculation of likelihoods for configurations with one polymorphic site and one fixed site. We deduce the final probability of both sites being fixed by subtracting all other probabilities from 1.

Given this table for a fixed sample size 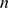 and fixed 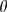, we can exactly infer an analogous table for smaller sample sizes and approximately infer a table for different values of 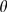.

A smaller sample size table can be directly generated for an arbitrary new sample size, 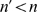; however, in the interests of clarity, we will describe how to generate a table when 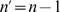, which can be iterated. Let the vector 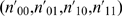 denote a configuration of sample size 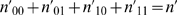. Assuming probabilities for ordered configurations (as generated by **complete** by default), the probability of this new configuration is the sum of the probabilities of 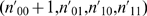, 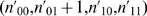, 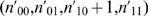 and 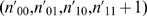.

Adjusting the table for a different 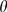 poses a greater challenge. One option would be to run **complete** many times to generate tables for different values of 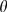, but this would be extremely time-consuming. Our alternative solution takes advantage of the fact that, while 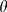 strongly affects the relative probabilities among the three broad categories of (both-sites-polymorphic, one-site-fixed, both-sites-fixed), 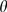 only mildly affects the relative probabilities of different configurations within these categories. The approximate probability of a site being polymorphic under the finite sites mutation model in a sample of size 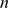 is 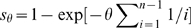 (approximate in the sense that this ignores the slight possibility of a site being polymorphic but having back mutations erase all traces of that polymorphism). If two sites are independent 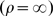, then the probabilities corresponding to these three categories of pairs are 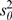, 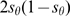, 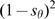. Now we assume that the ratio between the probabilities of these categories is independent of 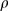 and approximate the probabilities of configurations under some new 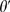 by multiplying by 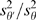 (if both sites are polymorphic) or 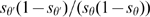 (if one site is fixed). If both sites are fixed, then we again deduce the probability by subtracting all other probabilities from 1.

Given these tabulated (or calculated) values, we use linear interpolation to arrive at the final probability for a given 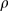. Linear interpolation as well as our numerical maximization algorithm require that the likelihood surface be reasonably smooth. The importance sampling algorithm leaves a small amount of error in its estimate of the likelihood, which can lead to small wiggles in the likelihood surface. We solve this problem by smoothing the tabulated values where necessary via cubic splines. Also, for configurations with a single fixed site, the importance sampling algorithm did not reduce the variance in the likelihood below the very low level of the slope across 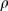, leading to numerical difficulties performing maximization on a non-smooth likelihood surface. We avoid this problem by making the likelihoods for these configurations constant across 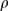 by setting them equal to their average value.

### Complexity and approximations

As alluded to earlier, a brute force implementation of the four nested loops in the composite likelihood function would take 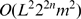 time where 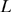 is the length of the assembly (or region of interest), 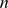 is the read depth and 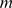 is the average number of missing nucleotides at each site, assuming a constant read depth. Real metagenomic data have variable read depth, which makes the situation even worse with the sequencing error component (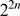) dominating the complexity at high-depth sites (i.e. where 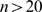). Instead we make several approximations:

1. Reduce amount of low quality data. We allow no more than five bases with quality below 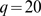 (1 in 100 chance of error) in any pair of sites. For an average read depth of 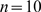 and a quality distribution from Sanger sequencing, this cutoff eliminates ∼3% of our lowest-information-content data for a significant speed increase.
2. Skip nearby pairs of sites. We consider only those pairs separated by at least 10 bases (in (1), change the second product to start at 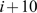) and we only make pairs for every 5th site (in (1), change the first product to take values 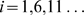). Any given pair of adjacent sites is highly unlikely to have had a recombination breakpoint between them. If the sites are separated by a greater distance, the chance of a recombination breakpoint between them increases. Thus this approximation sacrifices a small amount of information to reduce the overall number of pairs of sites. Empirically, simulations suggest this approximation does not greatly increase the variance of 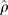.
3. Only use pairs of sites spanned by at least one chromosome (i.e. using the statistic defined in the next section, 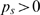). Pairs of sites not meeting this criteria tend to be far apart and contain relatively little information.
4. When accounting for error, only consider plausible true configurations, instead of all possible configurations. For a given pair of sites, we first sort the quality scores in ascending order (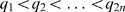). Then we iterate over truths in decreasing order of probability (for one error: 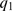, then 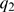, etc.; for two errors: 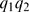, then 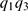, etc.) until the probability is less than 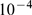 times as likely as the most probable configuration.

Given these approximations, a standard desktop computer can perform this estimation for 10 kb of sequence, average depth of 10 and a realistic error distribution in less than one hour.

### *Ρ_s_* statistic

Before we discuss our results, we need to quantify the amount of missing data between a given pair of sites. Define 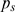 to be the proportion of chromosomes that span a particular pair of sites: 
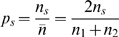
, where 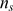 is the number of chromosomes spanning both sites (i.e. both sites are covered either by the same read or by paired reads) and 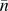 is the average number of chromosomes covering each site separately (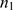 and 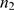, respectively).

The average value of this statistic together with the average sample size provide an indirect measure for the amount of information about recombination captured by pairs of sites within a given dataset.

### Sludge data

We applied our technique to the first 500 kb of the assembly of Candidatus *Accumulibacter phosphatis* from a recent metagenomic sequencing project of activated sludge from a wastewater treatment plant [13]. The sludge we analyzed came from a laboratory bioreactor in Madison, Wisconsin that had been seeded from a local wastewater treatment plant. We received the data (P. Hugenholtz, personal communication) in the form of a finished assembly consisting of ACE and PhD files covering a 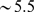 megabase scaffold of average depth 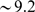. Equivalent data in a different form are also available directly from the Joint Genome Institute via the IMG/M system [25] and the NCBI Trace Archive (genome project id 17657).

## Results

**Figure 2:**
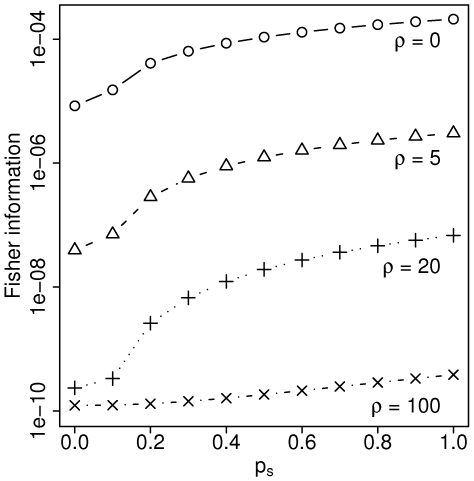
Information about 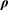 as a function of missing data. Fisher information for a single pair of sites of depth 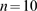, with varying amount of missing data as quantified by 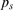 (0 = no chromosomes span both sites; 1 = all chromosomes span both sites) for different values of 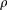.

We first investigate the information content of a single pair of sites as a function of the amount of missing data. This information sets an upper bound on our estimator's performance since we use the composite likelihood instead of the true likelihood. In particular, the Fisher information, 
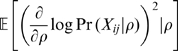
, for a single pair of sites with depth 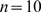 decreases with 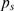, although the information only falls off dramatically for 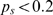 (Figure 2). We find these results encouraging since the average 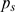 of pairs in the actual sludge metagenome falls just above this threshold at 0.21. Note that the Fisher information holds little meaning on an absolute scale since we calculate the information for a single pair of sites rather than for our actual data with many dependent pairs. Instead, the values in Figure 2 should be interpreted on a relative scale. For instance, for 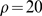, approximately ten independent pairs with 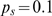 would contain the same information about 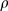 as a single pair with 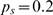.

The bulk of our analyses rely on simulated data where we know the truth and can evaluate the performance of our estimator. We use the program **ms** [26] in combination with **seq-gen** [27] to generate sequences across a 10 kb region under a finite-sites model of mutation (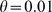 unless specified otherwise) and the coalescent with recombination. We simulate recombination as gene conversion with mean tract length fixed at 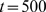 (see equation 2). The sample size (i.e. number of simulated chromosomes) is 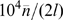 where 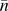 is the average read depth and 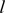 is the length of each read in a read-pair. We transform these sequences into metagenomic-style data by randomly distributing read starts uniformly across the simulated region and trimming each simulated sequence to only be present for the length of three segments: one read, the gap between read pairs, and one read. Our simulation assumes no variation in read length or distance between read pairs. Note that a gap of zero produces the same effect as unpaired reads with double the read length. For results with sequencing error, we assign quality scores from the true Sanger sequencing quality score distribution as determined from the sludge data. A “sequencing error” causes a switch from the true nucleotide to each of the other three with probability 1/3. Given that we are simulating relatively small datasets with low information content, we occasionally generate an assembly with a maximum likelihood at 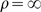. We exclude these values from all further analyses, but, for each parameter set, we report the proportion of replicates that yielded infinite parameter estimates either in Table 1 or in the text below.

**Table 1:** Proportion of simulation replicates with 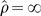 for each parameter set.

Parameters	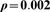	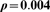	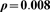	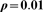	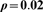	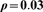	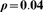
0.01,75,0	0.020	0.0040	0.0040	0.0040	0.0060	0.0020	0.004
0.01,500,0	0.026	0.0160	0.0080	0.0140	0.0020	0.0060	0.004
0.01,500,100	0.030	0.0140	0.0080	0.0140	0.0020	0.0120	0.012
0.01,500,500	0.026	0.0180	0.0060	0.0160	0.0080	0.0020	0.012
0.01,1000,0	0.022	0.0065	0.0045	0.0022	0.0046	0.0067	0.011
0.01,500,0*	0.016	0.0040	0.0000	0.0000	0.0000	0.0000	0.000
0.01,5000,0*	0.000	0.0000	0.0000	0.0000	0.0000	0.0000	0.004

First column lists parameters: 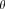, read length, gap between paired end reads. Asterisk (*) signifies simulations without sequencing error.

**Figure 3:**
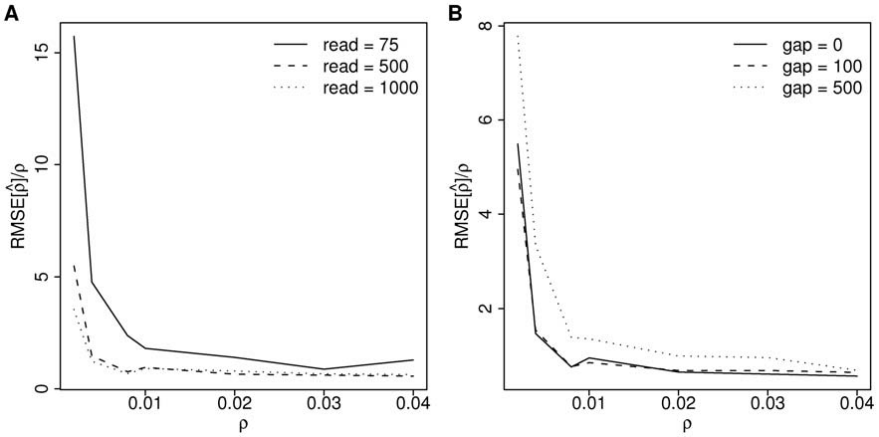
Performance of 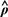. Root mean squared error (RMSE) of 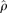 relative to the true 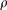 for paired-end reads with (A) different read lengths with gap = 0 separating the pairs and (B) different gap lengths with read length = 500. RMSE calculated from 500 replicate simulations of assembly size 10 kb, 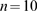, 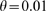, Sanger-distributed sequencing error.

We analyzed the performance of our estimator in the presence of sequencing error across a range of plausible values of 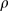 (0.002 to 0.04), read lengths roughly corresponding to current Illumina, 454 and Sanger sequencing technologies (75, 500, 1000) and gaps between read-pairs (0, 100, 500) by calculating the root mean squared error (RMSE) relative to the true value (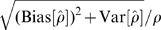; Figure 3). Note that while RMSE conveniently summarizes our estimator's sampling distribution, it obscures the inherent asymmetry of the distribution caused by the constraint 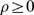. A clear trend emerges with lower relative RMSE accompanying increased recombination. The estimator has little bias (results not shown) and, for 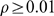, we are able to reliably estimate within a factor of 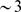 of the true value. For most parameters, increasing the read length reduces the variance by virtue of increasing 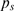, but for larger 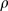 the results for 1 kb reads appear slightly worse than for 0.5 kb reads. Increasing the gap between the paired-end reads increases the variance for all except the very smallest 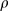. Intuitively, this makes sense: if all pairs of sites are very close together with low 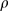 then a recombination event will only rarely occur between them; however, if all pairs are far apart with high 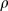 then recombination events will saturate between the pairs of sites.

**Figure 4:**
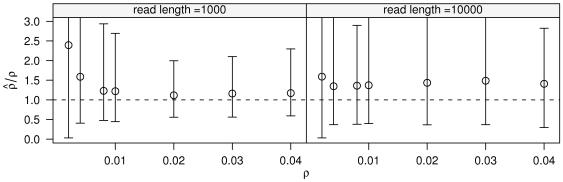
Metagenomic versus standard population sampling. Metagenomic data on left has 100 reads of 1 kb each; standard data on right has 10 reads of 10 kb each. Circles correspond to mean; whiskers show 2.5% and 97.5% percentiles for 250 replicate simulations of assembly size 10 kb, 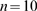, 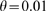, no read pairs, no sequencing error.

With the above results suggesting that longer read lengths do not always yield a better estimate, we decided to directly compare a metagenomic-style sample to a “standard” population genetic sample in which the same individuals are sequenced at all loci. The fair comparison keeps the total number of sequenced bases constant, so we simulate a 10 kb region with either 100 reads of 1 kb each or 10 reads of 10 kb each (Figure 4). For simplicity, we do not simulate sequencing error. As analyzed in the Discussion, despite the average depth being identical between the two sets of simulations, the metagenomic sample (on the left) exhibits less bias and much lower variance than the standard sample (on the right).

**Figure 5:**
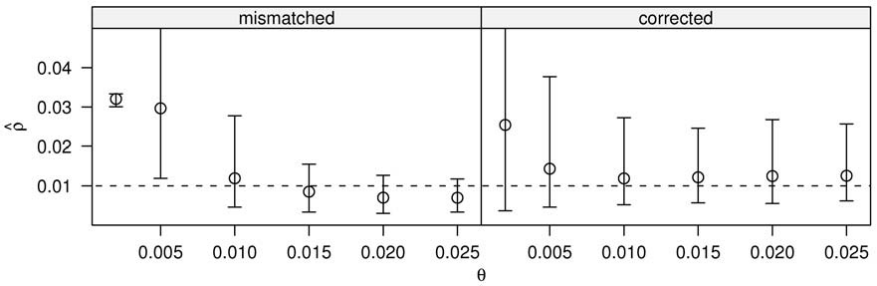
Likelihood correction for different *θ*s. All simulations use 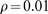 (dashed horizontal line) and estimates use likelihood table created for 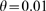. Left panel makes no adjustment for mismatch between the table 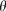 and the true 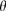. The odd variance for 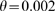 stems from 98% of simulations yielding infinite estimates and the remaining estimates being highly biased. Right panel uses the correction described in the Methods section. Circles correspond to mean; whiskers show 2.5% and 97.5% percentiles for 250 replicate simulations of assembly size 10 kb, 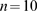, read length 1000, no read pairs, no sequencing error.

Next we tested our approximation that adjusts the two-site likelihoods for different values of 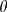 (see Methods subsection “Two-locus complete likelihoods without error”) by fixing 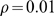 and simulating across 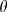 ranging from 0.002 to 0.025 while estimating 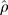 using a two-site likelihood table generated for 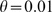 (Figure 5). Again we do not simulate sequencing error to focus on the effects of 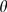. Here we see that the correction (on the right in Figure 5) works quite well for 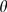 above the likelihood table's driving value (i.e. 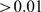) and somewhat less well for lower 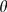, with 3% of the simulations for 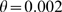 giving infinite estimates. However, the uncorrected estimator (on the left) is strongly biased, with 98% of simulations for 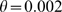 resulting in infinite (unplotted) estimates and 26% of those for 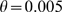. No other parameter values yielded any infinite estimates. The low 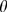 results are exacerbated by the correlation of 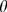 with the number of polymorphic sites. Lower 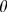 means fewer polymorphic sites; since the majority of information about recombination rate comes from polymorphic sites, we see a larger variance in our estimate of 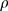 for low 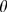.

**Figure 6:**
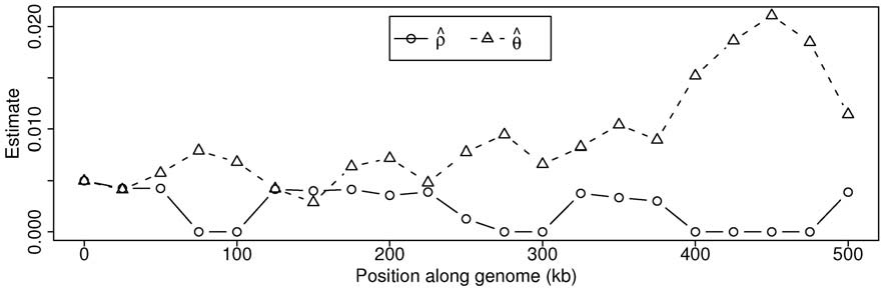
Parameter estimates from sludge data. Estimates generated by sliding 50 kb window in steps of 25 kb across first 500 kb of the sludge assembly.

Finally we apply our estimator to the sludge metagenomics project by sliding a 50 kb window in 25 kb steps across the first 500 kb of the assembly and independently estimating the recombination rate within each window (Figure 6). All windows produced finite estimates with 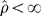.

## Discussion

The two-site composite likelihood estimator appears to be better suited for metagenomic samples (i.e. the purpose of this paper) than for standard population genetic samples (i.e. the purposes of [12],[19]) as seen from Figure 4. We believe this results from the balance of two opposing factors: greater linkage (less missing data) pushes the advantage toward the standard sample, while a larger genealogy with more independence pushes the advantage back toward the metagenomic sample. For the parameter ranges investigated here, the latter force wins and we see that the estimates for metagenomic samples have both less bias and lower variance for a fixed amount of sequencing. This result makes sense given the nature of the composite likelihood technique in which we treat each pair of sites as though it were independent of every other pair. The more chromosomes that are sampled, the more closely this independence assumption matches reality. An intriguing open question is how the composite likelihood estimator on metagenomic data compares to a full likelihood estimator on standard data, but we do not pursue this topic here.

The bias in the standard sample estimates (Figure 4) surprised us given theoretical results that assert consistency for the composite likelihood estimator [28]. However, consistency is an asymptotic feature and does not necessarily hold for finite samples. Indeed, further simulations of standard samples with greater sample depth reduced the bias to essentially zero with depth 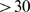 (results not shown). Given that metagenomic samples appear nearly unbiased with depth 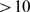, the added independence of the metagenomic sample must allow the estimator to converge faster toward the asymptotic results.

Further, in contrast to Hudson's and McVean's programs (**maxhap** and **LDhat**, respectively), our method makes use of all pairs of sites, including sites observed to be fixed. We include these sites primarily as a byproduct of properly accounting for sequencing error, but these additional data also help reduce our variance. As a bonus, using all sites automatically makes our pairwise likelihoods true likelihoods, thus fulfilling one of the requirements for Fearnhead's [28] results proving the consistency of the composite likelihood estimator. If fixed sites were not included, then the pairwise likelihoods would need to be made conditional on only using pairs of segregating sites, which becomes computationally challenging when dealing with missing data. In fact, while **maxhap** and **LDhat** allow missing entries in their input data, this feature is not described in the accompanying papers [12],[19], and these implementations do not properly condition their likelihoods to account for the fact that they only use segregating sites. The only disadvantage of using all pairs of sites is that the likelihood calculation scales linearly with the number of pairs and thus using all pairs takes longer; however, our implementation still runs in a reasonable amount of time on realistic amounts of data (see “Complexity and approximations” subsection in Methods).

Real data include sequencing errors, which have the potential to bias population genetic inference and increase the variance of estimators [29]. Trimming the data based on quality scores will help reduce these problems, but the remaining error must still be taken into account. We do not have analytic theory quantifying the amount of bias introduced by sequencing error, but simulations show that unaccounted-for errors produce estimates biased toward a specific finite value of 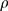 that depends on the read length and gap size (results not shown). Intuitively, sequencing error primarily produces singletons, which yield different configurations depending on the distance separating the two sites with errors. If the two sites are close together, then errors will tend to generate 01 and 10 states. If the two sites are far apart, then errors will tend to generate 1? and ?1 states. The first group of states (01, 10) provides evidence for higher recombination since, if both mutations originally fell on the same chromosome (state 11), then recombination would have been necessary to break them up to be (01, 10). The second group of states (1?, ?1) provides evidence for lower recombination since this pattern of missing data is more likely to have arisen from (11, 11) states, which is suggestive of no recombination, then (01, 10) states. Thus sequencing error introduces a highly artificial pattern of configurations, with a combination of evidence for high recombination between close pairs of sites and low recombination between distant pairs of sites leading to a maximum likelihood at an intermediate value. For paired-end reads of 500 bases separated by a gap of 0, errors drive toward 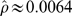.

**Figure 7:**
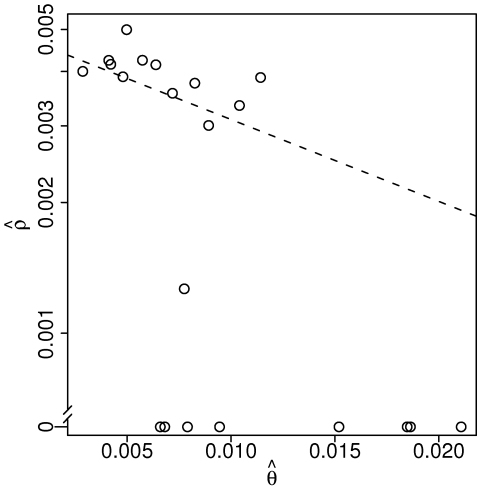
Log-linear relationship between 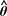 and 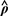. Circles are data from Figure 6, dashed line is log-linear regression using non-zero values of 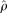.

The striking inverse correlation between the estimates of 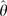 and 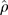 from the sludge data (Figure 6) could either be the result of an unknown artifact or a biological reality stemming from a dependence between recombination efficiency and sequence divergence. One possibility for an artifact would be sequencing error not accounted-for in the quality scores (e.g. a PCR error before sequencing). Such errors would certainly lead to increased estimates of 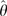, but, on the basis of our simulations, seem unlikely to drive 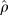 down to 0. Also, such errors would have to occur non-uniformly across the genome at a granularity of 50 kb, which seems implausible. Another potential source for an artifact is our two-step estimation procedure in which we first estimate 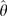 without regard to recombination and then estimate 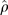 conditional on 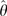. Again, however, simulations reveal that, while 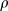 affects the variance of 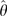, the estimator is unbiased across all tested 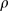 and shows no correlation between 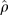 and 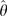 (results not shown). Without a clear artefactual explanation, we turn toward biology. Laboratory experiments have shown a negative log-linear dependence between sequence divergence and transformation efficiency [30], and an analysis of a different metagenomic dataset found a similar dependence between divergence and parsimoniously-inferred recombination events [10]. Our data suggest that this pattern holds at a finer resolution with subtle increases in diversity, as quantified by 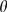, leading to lower rates of recombination in a log-linear manner, with the exception of regions in which recombination appears nonexistent (Figure 7).

On an absolute scale, these estimates from the sludge data fall into a plausible range for bacterial populations. For instance, in *Campylobacter jejuni* 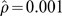 [31] and in *Neisseria meningitidis* 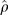 ranges from 0.00270 to 0.034 [11]. However, previous estimates of microbial recombination rates have been based on much smaller amounts of data (in these examples, 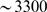 bases) relative to the sludge windows of 50 kilobases. In addition, *C. jejuni* and *N. meningitidis* are both pathogens, which makes for a quite different ecological and evolutionary environment than that of the nonpathogenic sludge bacterium *A. phosphatis*. When the sludge estimates of mutation and recombination are viewed relative to each other, we see that mutation events generally occur more frequently than recombination events (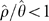), which places *A. phosphatis* more toward the clonal end of the bacterial spectrum [32].

Overall, our new estimator produces surprisingly accurate estimates of recombination rate, particularly considering the amount of missing data. The real power of the estimator derives from the greater independence of the genealogies underlying the sample; sequencing error and missing data present hurdles to accessing this information but our estimator has surmounted them. Despite our motivation from microbial populations, our method itself makes no assumptions inherent to microbial populations. For our purpose, a “metagenomic” sample simply means sampling a mixture of a large number of individuals from a single species, in which each read (or pair of reads) can be safely assumed to have originated from a different individual. Given the results from the comparison to a standard sample, the metagenomic approach should always be followed to obtain maximal information about recombination for a fixed amount of sequencing.

An implementation of our Population genetic Inference In Metagenomics (PIIM) method is freely available for download from http://ib.berkeley.edu/labs/slatkin/software.html.

## References

[1] Lenski RE, Winkworth CL, Riley MA (2003). Rates of DNA sequence evolution in experimental populations of Escherichia coli during 20,000 generations.. J Mol Evol.

[2] Whitaker RJ, Grogan DW, Taylor JW (2005). Recombination shapes the natural population structure of the hyperthermophilic archaeon Sulfolobus islandicus.. Mol Biol Evol.

[3] Myers S, Bottolo L, Freeman C, McVean G, Donnelly P (2005). A fine-scale map of recombination rates and hotspots across the human genome.. Science.

[4] Smith GR (1994). Hotspots of homologous recombination.. Experientia.

[5] Brouns SJ, Jore MM, Lundgren M, Westra ER, Slijkhuis RJ (2008). Small CRISPR RNAs guide antiviral defense in prokaryotes.. Science.

[6] Gevers D, Cohan FM, Lawrence JG, Spratt BG, Coenye T (2005). Opinion: Re-evaluating prokaryotic species.. Nat Rev Microbiol.

[7] Fraser C, Hanage WP, Spratt BG (2005). Neutral microepidemic evolution of bacterial pathogens.. Proc Natl Acad Sci U S A.

[8] Smith JM, Smith NH, O'Rourke M, Spratt BG (1993). How clonal are bacteria?. Proc Natl Acad Sci U S A.

[9] Didelot X, Falush D (2007). Inference of bacterial microevolution using multilocus sequence data.. Genetics.

[10] Eppley JM, Tyson GW, Getz WM, Banfield JF (2007). Genetic exchange across a species boundary in the archaeal genus ferroplasma.. Genetics.

[11] Jolley KA, Wilson DJ, Kriz P, McVean G, Maiden MC (2005). The influence of mutation, recombination, population history, and selection on patterns of genetic diversity in Neisseria meningitidis.. Mol Biol Evol.

[12] McVean G, Awadalla P, Fearnhead P (2002). A coalescent-based method for detecting and estimating recombination from gene sequences.. Genetics.

[13] Martn HG, Ivanova N, Kunin V, Warnecke F, Barry KW (2006). Metagenomic analysis of two enhanced biological phosphorus removal (EBPR) sludge communities.. Nat Biotechnol.

[14] Tyson G, Chapman J, Hugenholtz P, Allen E, Ram R (2004). Community structure and metabolism through reconstruction of microbial genomes from the environment.. Nature.

[15] Johnson PLF, Slatkin M (2006). Inference of population genetic parameters in metagenomics: a clean look at messy data.. Genome Res.

[16] Simmons SL, Dibartolo G, Denef VJ, Goltsman DS, Thelen MP (2008). Population genomic analysis of strain variation in Leptospirillum group II bacteria involved in acid mine drainage formation.. PLoS Biol.

[17] Stumpf MP, McVean GA (2003). Estimating recombination rates from population-genetic data.. Nat Rev Genet.

[18] Wang Y, Rannala B (2009). Population genomic inference of recombination rates and hotspots.. Proc Natl Acad Sci U S A.

[19] Hudson RR (2001). Two-locus sampling distributions and their application.. Genetics.

[20] Wall JD (2004). Estimating recombination rates using three-site likelihoods.. Genetics.

[21] Ewing B, Green P (1998). Base-calling of automated sequencer traces using phred. II. Error probabilities.. Genome Res.

[22] Frisse L, Hudson RR, Bartoszewicz A, Wall JD, Donfack J (2001). Gene conversion and different population histories may explain the contrast between polymorphism and linkage disequilibrium levels.. Am J Hum Genet.

[23] Langley CH, Lazzaro BP, Phillips W, Heikkinen E, Braverman JM (2000). Linkage disequilibria and the site frequency spectra in the su(s) and su(w(a)) regions of the Drosophila melanogaster X chromosome.. Genetics.

[24] Fearnhead P, Donnelly P (2001). Estimating recombination rates from population genetic data.. Genetics.

[25] Markowitz VM, Ivanova NN, Szeto E, Palaniappan K, Chu K (2008). IMG/M: a data management and analysis system for metagenomes.. Nucleic Acids Res.

[26] Hudson RR (2002). Generating samples under a Wright-Fisher neutral model of genetic variation.. Bioinformatics.

[27] Rambaut A, Grassly NC (1997). Seq-Gen: an application for the Monte Carlo simulation of DNA sequence evolution along phylogenetic trees.. Comput Appl Biosci.

[28] Fearnhead P (2003). Consistency of estimators of the population-scaled recombination rate.. Theor Popul Biol.

[29] Johnson PLF, Slatkin M (2008). Accounting for bias from sequencing error in population genetic estimates.. Mol Biol Evol.

[30] Roberts MS, Cohan FM (1993). The effect of DNA-sequence divergence on sexual isolation in Bacillus.. Genetics.

[31] Wilson DJ, Gabriel E, Leatherbarrow AJ, Cheesbrough J, Gee S (2009). Rapid evolution and the importance of recombination to the gastroenteric pathogen Campylobacter jejuni.. Mol Biol Evol.

[32] Hanage WP, Fraser C, Spratt BG (2006). The impact of homologous recombination on the generation of diversity in bacteria.. J Theor Biol.

